# Supplementary material for: Does managerial myopia promote enterprises over-financialization? Evidence from listed firms in China
Source: PLoS One. 2024 Sep 5;19(9):e0309140. doi: 10.1371/journal.pone.0309140 (PMC11376576; doi:10.1371/journal.pone.0309140)
Supplement: Attachment — Submitted filename: Response to reviewers.doc [file pone.0309140.s001.doc]

**Responses to editor of PONE: regarding the paper**

''Does managerial myopia promote enterprises over-financialization? Evidence from listed firms in China'' (Ref: PONE-D-24-06837),

**by Yanchi Chen, Ju Ye and Qi Shi**

Dear Editor:

On behalf of all co-authors, I would like to thank you and the reviewers for your serious and thorough work in refereeing this paper. Thank you for giving us a chance to revise and improve the quality of our article.

We have read the reviewers’ and your comments carefully and have made revision which marked in red in the paper. We have tried our best to revise our manuscript according to all suggestions.

Attached please find the revised version, which we hope that you will find this updated manuscript to your satisfaction and consider it for publication as an article in *PLOS ONE*. A point-by-point response to the reviewers’ comments and concerns is given below.

Thank you for taking the time to consider our research and we look forward to hearing from you at your earliest convenience

Yours sincerely,

Corresponding author

Ju YE

Institute of Food and Strategic Reserves

Nanjing University of Finance and Economics

Nanjing, 210023, P. R. China

E-mail: c774402944@126.com

**Reply to editor and each reviewers’ comments**

**Editor:**

1. Please ensure that your manuscript meets PLOS ONE’s style requirements, including those for file naming.

**Response:** Thank you very much for your thoughtfulness in providing us with links to the style template websites. We have carefully reviewed the guidelines provided by PLOS ONE and have modified the formatting of headings, tables, formula fonts, references, etc. to ensure that our manuscript meets requirements. If you have any further suggestions or specific requests regarding the style or format of our manuscript, please feel free to let us know. We are committed to following all guidelines set forth by PLOS ONE to ensure a smooth and efficient review process.

2. Please state what role the funders took in the study. If the funders had no role, please state: “The funders had no role in study design, data collection and analysis, decision to publish, or preparation of the manuscript.” If this statement is not correct you must amend it as needed.

**Response and revision**: The role of funders in research is as follows: “This work was supported by the [National Social Science Fund of China] under Grant [22VRC007], which is led by Professor QiShi, with Mr. Yeju serving as one of the primary contributors to this project; [Institute of Food and Strategic Reserves, Nanjing University of Finance and Economics] under Grant [ BSZX2023-07], which is led by Miss. Yanchi Chen. The funders had no role in study design, data collection and analysis, decision to publish, or preparation of the manuscript.”

**Reviewer #1:**

1. Literature review section needs optimization, lacks research on the impact of managerial myopia on over-financialization in enterprises.

**Response and revision:** According to the reviewer’s comment, we have optimized literature review section to make it more organized. We supplemented the literature on managerial myopia regarding financialization and summarized the existing literature as follows: “The relevant literature on the impact of managerial myopia on excessive corporate financialization mainly focuses on the effects of corporate financialization and its underlying logic, as well as the measurement of managerial myopia and influencing factors. Concerns about the impact of corporate financialization are mainly concentrated in the macroeconomic domain among scholars.” And we elaborated on the existing research gaps and the rationale behind our study of this issue to better articulate the viewpoints presented in our paper as follows: “a fundamental contradiction between micro-level enterprises and macroeconomic financialization is the widespread financialization of enterprises, in which the degree of financialization of enterprises does not match the level of their own resource management, resulting in the phenomenon of the crowding-out of enterprises' investment in fixed assets from "real to virtual". It must be admitted that financialization represents a kind of financial investment behavior driven by enterprise resource management, and enterprise financialization is also conducive to enterprise development to a certain extent. Therefore, compared with the financialization behavior caused by managers' short-sightedness, the study believes that it is more important to prevent the excessive financialization behavior caused by managers’ short-sightedness.”. Lastly, we added some additional literature to better articulate the viewpoints of this paper.

2. It is suggested to summarize the contribution of the article and placed it in the first part.

**Response and revision:** According to the reviewers’ comments, we have summarized the marginal contributions of this paper in the first part as follows: “The marginal contributions of this study are as follows: Firstly, we are among the first to construct an indicator of excessive financialization based on the perspective of corporate financial heterogeneity. This enriches research on corporate financialization, helping scholars to correctly grasp the dual nature of financialization and avoid its negative effects. Secondly, we consider the differences between managerial myopia and corporate financialization, empirically testing the phenomenon of excessive financialization caused by managerial myopia, helping shareholders better understand the underlying motives of financialization and achieve a balance between short-term and long-term interests. Finally, the study also considers, through moderation effect models, the impact of managerial myopia on corporate excessive financialization under external conditions such as financing constraints and financial distress, providing a theoretical basis for better corporate development.” Given the extensive content of the introduction section, to facilitate readers in better observing the marginal contributions of this paper, we have made deletions and adjustments to the structure of the first section, making the article more organized.

3. Please read the entire article carefully and correct some details. For example, the fonts in Table 4 are not consistent.

**Response and revision:** We appreciate the careful comments provided by the reviewers. In addition to addressing the detailed issues pointed out by the reviewers, we have reorganized the entire manuscript and conducted proofreading, rectifying inconsistencies in some sentences and inconsistencies in font sizes. For example, the fonts in Table 4 and in-text formula size have been modified; add note as follows: “Note: Standard errors are in brackets, ***,**,* indicate significant at 1%, 5%, and 10% level of significance, respectively.” below result tables.

4. The logical hierarchy of the article's structure is somewhat redundant and needs adjustment. Both the literature review in the second section and the research methodology in the third section provide definitions of "managerial myopia" and "over-financialization in enterprises." It is suggested to consolidate and optimize these definitions.

**Response and revision:** In response to the reviewers’ suggestions, we have deleted redundant definitions of concepts in the third section and integrated them uniformly into the second section as follows. Additionally, we have streamlined the definition of relevant concepts in the first section to enhance the logical coherence and organization of the article's structure.

5. The robustness check section lacks textual description, failing to elucidate how the results of the robustness check specifically contribute.

**Response and revision:** In the empirical analysis section, we have supplemented explanations regarding robustness tests to elucidate the rationality of applying robustness test methods in this study. For eliminating the impact of the financial crisis, we added the realistic reasons for the selection at the beginning as follows: “The root causes of the 2008 global financial crisis stemmed from improper real estate financial policies and the misuse of financial derivatives, which clearly fueled excessive financialization behavior in enterprises. After the financial crisis of 2008, some enterprises learned from experience, leading to a moderation in the situation of excessive financialization.” and textual description as follows: “The results indicate that even after excluding the influence of financial crises, managerial myopia still promotes excessive financialization in enterprises.” For replacement of variable measure, we added the realistic reasons for the selection at the beginning as follows: “The substitution of explanatory variables and dependent variables is a commonly used method in robustness testing, aiming to examine whether the causal relationship between variables still holds.” and textual description as follows: “The conclusion that managerial myopia increases financial asset allocation is consistent with the findings of [44].” For endogeneity test, we added the realistic reasons for the selection at the beginning as follows: “Causal inference should exclude bidirectional causality between independent and dependent variables, meaning that short-term gains from corporate financialization may lead to managerial overconfidence, making them more myopic.”

6. Other papers related to green low-carbon endogenous economic growth can refer to:

Yueh-Hsia Huang, Lan Sun, Tyng-Bin Ger. An analysis of enterprise resource planning systems and key determinants using the Delphi method and an analytic hierarchy process[J]. Data Science in Finance and Economics, 2023, 3(2): 166-183. doi: 10.3934/DSFE.2023010

Li, Z., Chen, H., & Mo, B. (2023). Can digital finance promote urban innovation? Evidence from China. Borsa Istanbul Review, 23(2), 285-296. doi:10.1016/j.bir.2022.10.006

Yonghong Zhong, Junhao Zhong. The spread of debt risk from real estate companies to banks: Evidence from China[J]. Quantitative Finance and Economics, 2023, 7(3): 371-390. doi: 10.3934/QFE.2023018

**Response and revision:** We appreciate the literature provided by the reviewers, which has been helpful in enhancing our argumentation. We have incorporated these references into the literature review section as follows: “In China, the real estate industry, which has a high degree of financialization, also faces the negative impact of debt risk shifting to banks, which is worthy of social and government vigilance [19]…Furthermore, corporate finance exhibits externalities, promoting innovation levels by alleviating financing constraints for other enterprises [21]…Effective resource planning and management are important pathways for enterprises to achieve their expected goals [22].”

**Reviewer #2:**

1.The authors should provide a clear explanation of the study's scope and objectives, substantiated by relevant studies. Additionally, the literature review appears weak and lacks cohesion.

**Response and revision:** Firstly, we optimized the structure of the first section to prevent excessive financialization from being the main focus, entering into it in the third paragraph of the first section to clearly state the research objectives and scope as follows: “This paper wants to emphasize that enterprise financialization behavior is not equivalent to managerial myopia…Therefore, the financialization behavior of enterprises may not necessarily harm the long-term sustainable development of enterprises. Thus, this paper takes Chinese non-financial industry listed companies from 2005 to 2022 as samples, quantifying managerial myopia through the analysis of annual report texts;” Secondly, we also optimized the structure of the second section, summarizing the scope of relevant literature, and added transitional sentences between paragraphs to help readers better understand the research content, like “The relevant literature on the impact of managerial myopia on excessive corporate financialization mainly focuses on the effects of corporate financialization and its underlying logic, as well as the measurement of managerial myopia and influencing factors. Concerns about the impact of corporate financialization are mainly concentrated in the macroeconomic domain among scholars.”, “Research on managerial myopia tends to focus on measurement methods, which can effectively reflect managers' behavioral tendencies.”, “Regarding the impact of managerial myopia on corporate financialization,”

2.The measurement methods should be justified and compared to other methods to explain why this specific method was chosen.

**Response and revision:** In the section on empirical model specification, we have added comparative explanations for the methods of excessive financialization as follows: “In this way, this method differs from Wang et al.'s calculation of excessive financialization based on financial risks [24], as it is more conducive for enterprises to focus on long-term development prospects.” Our measure of managerial myopia utilizes an established method from existing literature, which is relatively comprehensive, so we have added relevant explanations as follows: “In the field of managerial myopia, the method of capturing managerial strategic information through word frequency text has become relatively mature. Brochet et al. [25]…Hu et al. [Error: Reference source not found]”. As our empirical model undergoes model testing, we have not added additional explanations to ensure clarity of content.

3.The author should also justify the choice of statistical methods, sample size, and the application of the chosen tests to ensure their validity and correctness.

**Response and revision:** In the empirical analysis section, we have added explanatory on the choice of statistical methods as follows: “In this way, this method differs from Wang et al. (2023)’s calculation of excessive financialization based on financial risks, as it is more conducive for enterprises to focus on long-term development prospects.”, sample selection handling as follows: “textual analysis inclusion:…to ensure the completeness of the explanatory variable data; exclusion of specific types:…to avoid outliers in the sample;” , robust method selection as follows: “The substitution of explanatory variables and dependent variables is a commonly used method in robustness testing, aiming to examine whether the causal relationship between variables still holds.”, endogeneity methods as follows: “Causal inference should exclude bidirectional causality between independent and dependent variables, meaning that short-term gains from corporate financialization may lead to managerial overconfidence, making them more myopic.”, etc., to elucidate the effectiveness of the methods.

4.The author(s) should explain whether the study is in line with prior studies or not, also whether it supports any theories.

**Response and revision:** In the robustness testing section, we have added relevant literature on the impact of managerial myopia on financialization as follows: “The conclusion that managerial myopia increases financial asset allocation is consistent with the findings of [44].” to demonstrate the consistency and reliability of the conclusions of the article.

All suggestions have been accepted and the manuscript modified accordingly. We thank again the anonymous reviewers for their insightful comments and suggestions, which greatly help with our improvements.

Corresponding author

Ju YE

Institute of Food and Strategic Reserves

Nanjing University of Finance and Economics

Nanjing, 210023, P. R. China

E-mail: c774402944@126.com
